# Academic-related factors and daily lifestyle habits associated with adolescent idiopathic scoliosis: a case-control study

**DOI:** 10.1265/ehpm.22-00243

**Published:** 2023-04-13

**Authors:** Qiufen Dou, Zhixiang Zhu, Liwan Zhu, Wanxin Wang, Lan Guo, Shouhang Ru, Xiaosheng Chen, Lei Yang, Ciyong Lu, Bin Yan

**Affiliations:** 1 Department of Medical Statistics and Epidemiology, School of Public Health, Sun Yat-sen University, Guangzhou, China; 2 The First Affiliated Hospital of Shenzhen University, Shenzhen, China; 3 Department of Spine Surgery, The Shenzhen Second People’s Hospital, Shenzhen, China; 4 Shenzhen Youth Spine Health Center, Shenzhen, China

**Keywords:** Adolescent idiopathic scoliosis, Academic-related factors, Daily lifestyle habits

## Abstract

**Background:**

Adolescent idiopathic scoliosis (AIS) is the most prevalent spinal deformity, which may have long-term negative consequences on adolescents. The research on the etiology is of great importance for identifying high-risk population and formulate tailored prevention. This study aimed to evaluate the association between academic-related factors and daily lifestyle habits and AIS.

**Methods:**

In this population-based case-control study, 491 AIS cases and 1,346 healthy controls that frequency-matched by age and sex were recruited in Shenzhen, Southern China. AIS was diagnosed as a Cobb angle ≥ 10° on standing posteroanterior radiographs of the whole spine. The academic-related factors (e.g., reading and writing posture) and daily lifestyle habits (e.g., intake of milk and dairy products) were collected by a self-reported questionnaire. The logistic regression analysis was performed.

**Results:**

After adjusting for potential confounding factors, multivariable logistic regression models demonstrated that academic-related factors were associated with AIS. Individuals with poor reading and writing posture were more likely to have AIS (AOR: 2.06, 95%CI: 1.58–2.68). Moreover, there was a significant association between heavy school bags and AIS (AOR: 2.22, 95%CI: 1.50–3.31). Additionally, adolescents who reported daily screen time on weekdays over 2 hours were more likely to develop AIS (*P* < 0.001). Regarding daily lifestyle habits, individuals without the habit of taking milk and dairy products had a higher risk of developing AIS (AOR: 1.87, 95%CI: 1.29–2.71).

**Conclusions:**

Academic-related factors and daily lifestyle habits were associated with AIS among Chinese adolescents. Schools, families, and related facilities are recommended to take actions on developing effective prevention and management strategies that integrates “Student-Family-School-Education-Health-Sports” for AIS.

## Introduction

Adolescent idiopathic scoliosis (AIS) is defined as a complex three-dimensional deformity of spine, which involves the lateral curvature, posteroanterior deviation or axial rotation of one or more segments of the spine without any congenital spinal abnormalities or identifiable causes [1]. It mainly occurs during adolescence [2], and it is the most prevalent spinal deformity that seriously disturbs adolescents worldwide [3]. The global prevalence of AIS ranges from 1% to 3%, and girls are more likely to develop [4]. According to the monitoring data of the National Health Commission of the People’s Republic of China, the detection rate of abnormal spinal curvature among Chinese primary and secondary school students in 2019 was 2.8% [5]. In addition, the results of the population-based scoliosis screening program during 2018 and 2019 in Shenzhen showed that the estimated prevalence of AIS was 3.2% [6].

Patients with mild or moderate AIS are usually asymptomatic. Nevertheless, as the curvature progresses, severe spinal deformity and the resulting surgical treatment [3] may induce lasting negative physical [1, 7, 8], psychological [9, 10] and socioeconomic consequences [11, 12]. Therefore, early detection, diagnosis and treatments are necessary and the research on the etiology is of great importance for identifying high-risk population and formulate tailored prevention. To date, the etiology and pathogenesis of AIS remain unclear and are considered to be multifactorial [13]. Although genetic factor has been proven to play a crucial role in the onset of AIS, environmental and lifestyle-related factors were non-negligible, either [14, 15].

Adolescents are experiencing a special period of rapid physical development [16, 17], in which the musculoskeletal system was not fully mature and was susceptible to external environmental factors [18]. Furthermore, during this period, adolescents establish patterns of lifestyle that may insert long-term impacts on their physical and psychological development [19]. Academic-related factors is one of the most important lifestyle-related factors in adolescents’ daily life. School adolescents always spend a lot of time sitting at desks or in front of screens for learning. Chronically incorrect sitting posture and carrying overweight backpacks may contribute to the musculoskeletal disorders, such as changing the spine shape and causing low back pain [20, 21]. A study conducted among senior high school graduates in Haidian district of Beijing in 2015 reported that poor sitting posture increased the risk of AIS [22], which was consistent with the findings of Chen et al. [23]. Scaturro D et al. [24] assessed the relationships between the use of backpack and the daily meters traveled with a backpack and AIS, but found no significant association. Additionally, previous studies have indicated that adolescents with long-time sedentary behavior tend to have lower bone mineral density, which was proved to be a key determinant of osteoporosis or fracture risk [25–27].

In addition, previous studies have shown that daily lifestyle habits, such as dairy consumption, positively affects peak bone mass and strength that can reduce the risk of osteopenia, which has been widely reported in previous studies to be prevalent in patients with AIS [28–31]. In addition, scholars have found that sleep disorders can lead to imbalances in bone metabolism, leading to osteopenia and osteoporosis [32, 33]. Watanabe K, et al. [15] explored the correlations between daily lifestyle habits and AIS, but found no association.

The above evidence indicates that these factors may be important for bone health and development. However, the health effects of these factors on AIS are not fully understood and inconclusive. Therefore, we conducted this study to evaluate the associations between these modifiable academic-related factors, daily lifestyle habits and AIS, and to suggest targeted preventive strategies for high-risk populations.

## Materials and methods

### Study design and participants

**Fig. 1:**
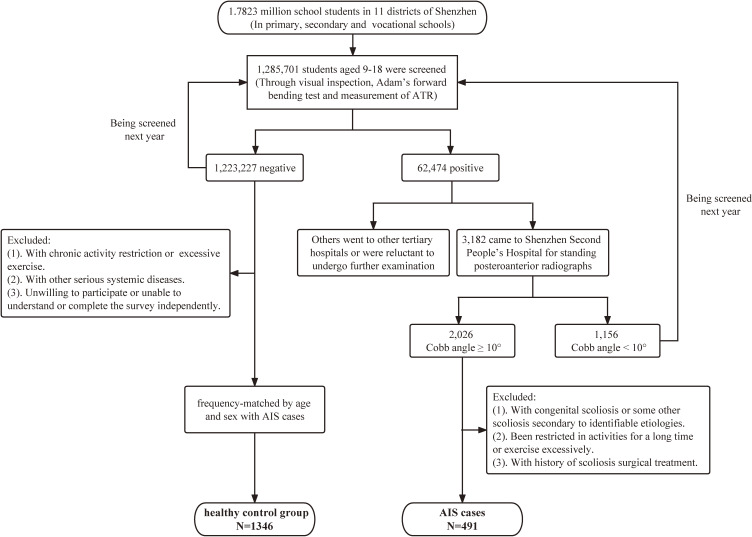
Study flow chart

A population-based matched case-control study was conducted based on the Chinese School-based Scoliosis Screening Program (CSSSP) in Shenzhen city, Guangdong Province, southern China, which was conducted and administered by the Shenzhen Youth Spine Health Center (SYSHC) of the Shenzhen Second People’s Hospital. From January 2021 to December 2021, the screening program was conducted by trained rehabilitation therapists from SYSHC among primary and secondary school students. The program covered students from the fourth grade (aged 9–10 years) of primary school to the third grade (aged 17–18 years) of high school in 11 districts of Shenzhen. Screening methods was conducted according to the national scoliosis screening standardized protocol (GB/T 16133-2014) [34] including visual inspection of physical signs, Adam’s forward bending test (FBT), and measurement of the angle of trunk rotation (ATR) with scoliometer. Individuals with an ATR > 5° and positive FBT or exhibiting one or more significant clinical signs in visual inspection, such as high and low shoulder, scapula tilt, pelvic tilt, and flat back were identified as positive and they would be referred to tertiary hospitals for standing posteroanterior radiographs of the whole spine for a definitive diagnosis. Individuals with negative screening results constituted the healthy control group (Fig. 1). More details about the screening procedures could be found in our previous published study [35].

### AIS case group

#### Inclusion criteria

Individuals were included in AIS case group if all of the following conditions were met: (1) aged 10 to 18 years; (2) diagnosed by profession spine surgeons based on the criteria of having a radiological lateral Cobb angle ≥ 10° and without any identifiable causes or underlying diseases [36]; (3) without other serious systemic diseases; (4) willing to participate and had the ability to understand and complete the survey independently.

#### Exclusion criteria

Individuals were excluded from AIS case group if they had any of the following: (1) with congenital scoliosis, neuromuscular scoliosis or some other scoliosis secondary to identifiable etiologies; (2) been restricted in activities for a long time or exercise excessively; and (3) history of scoliosis surgical treatment.

### Healthy control group

#### Inclusion criteria

Individuals were included in healthy control group if all of the following conditions were met: (1) with negative results in school screening; (2) frequency-matched by age and sex with AIS case group; (3) aged 10 to 18 years; (4) without other serious systemic diseases; (5) willing to participate and had the ability to understand and complete the survey independently.

#### Exclusion criteria

Individuals with chronic activity restriction or excessive exercise were excluded from healthy control group.

Finally, a total of 491 AIS cases and 1,346 healthy controls were included in this study. All the eligible participants were fully informed of the purpose of the survey and were invited to participate voluntarily. Most of the 491 AIS cases were newly diagnosed from January 2021 to December 2021. A self-administered questionnaire was used to investigate the factors possibly associated with AIS. The risk factors evaluated in our study were the behaviors of the past one year, which were assumed to be relatively stable and would not change easily, to some extent reducing the possibility of reverse causation. The questionnaires were administered by trained investigators in the classrooms without the presence of the teachers (to avoid any potential information bias). The cases and controls were assessed at the same period and all the participants completed the questionnaire by themselves. This study was conducted in accordance with the Declaration of Helsinki, and obtained ethical approval from the Shenzhen Second People’s Hospital Institutional Review Board (Ethics Number: 20211013002-fs01). After the study had been fully informed in detail, written or oral informed consent was obtained from each participating student and their parents or legal guardians.

### Measures

#### Demographic variables

Socio-demographic and general characteristics were collected by self-reported questionnaire, include age, gender, family history of AIS, living arrangement (1 = live in school dormitory, 2 = live at home), academic pressure (1 = none, 2 = low, 3 = high), enrollment pressure (1 = none, 2 = low, 3 = high), classmate relationships (1 = good, 2 = average, 3 = poor) and teacher-classmate relationships (1 = good, 2 = average, 3 = poor). The anthropometric variables assessed in the study including height, weight and body mass index (BMI). BMI was calculated by dividing self-reported weight in kilograms by the square of self-reported height in meters.

#### Academic-related factors

Academic-related factors include myopia (1 = not, 2 = yes), reading and writing posture (1 = good: sitting upright with a distance of more than 30 centimeters between the eyes and book, 2 = poor: the distance between the eyes and book was less than 30 centimeters and leaning one’s body when reading and writing), dominant hand when writing (1 = left, 2 = right), cross your legs (1 = not, 2 = yes), daily screen time on weekdays (1 = “≤ 2 h”, 2 = “> 2 h”) [37], mode of transportation of backpacks (1 = with both handles of the backpack on each shoulder, 2 = with one of the handles on a shoulder or by a hand, and 3 = others), weight of the school bags (1 = light, 2 = medium, and 3 = heavy). The weight of the school bags was evaluated by asking participates “How do you feel about the weight of your school bag”. The evaluation of all these factors was based on self-reported information from the participants.

#### Daily lifestyle habits

The daily lifestyle habits we collected mainly included intake of milk and dairy products, sleeping posture and sleep quality. Milk and dairy products intake was evaluated by asking participates “Did you have the habit of intake of milk and dairy products during last year” (1 = not, 2 = yes). Sleeping posture was assessed based on the participants’ self-reported posture when sleeping in bed (the answer was coded as 1 = stretch, 2 = curl up, 3 = unclear). Sleep quality was measured by the Chinese version of the Pittsburgh Sleep Quality Index (PSQI), which has been validated and widely used among Chinese adolescents [38]. The Chinese version of PSQI consists of 19 items containing seven components: subjective sleep quality, sleep latency, sleep duration, habitual sleep efficiency, sleep disturbance, sleep medication use, and daytime dysfunction. The score for each component ranges from 0 to 3. The global PSQI score consists of the summation of all seven components and ranges from 0 to 21. According to the global PSQI score, the sleep quality was categorized into “very good (0–5)”, “okay (6–10)”, “average (11–15)” or “poor (16–21)” [39, 40].

### Statistical analysis

Descriptive analyses were conducted to describe the demographic characteristics of cases and controls, and the data were expressed as number (%) and mean (standard deviation, SD). To compare the differences between the AIS cases and healthy control groups, Chi-square test for categorical variables and *t*-test for continuous variables were performed. The univariable and multivariable logistic regression analysis were used to estimate the degree of association between the related factors and AIS. All Data were analyzed using SPSS version 25.0 (IBM Corp, Armonk, NY, USA), and *P*-value less than 0.05 was considered statistically significant (tested two-sided).

## Results

### Demographic characteristics of all participants

The demographic characteristics of the 1,837 participants are shown in Table 1. The case group and the control group were frequency-matched according to age (*P* = 0.178) and sex (*P* = 0.970). The mean age of the case and control groups were 13.69 (SD: 1.79) and 13.81 (SD: 1.72) years, respectively. The percentage of male participants were 27.7% and 27.8% in the case and control groups. In addition, for AIS cases, the mean of BMI (SD) was 18.40 (2.80) kg/m^2^, whereas the mean of BMI (SD) was 19.92 (3.49) kg/m^2^ for the controls, the difference between them was statistically significant (*P* < 0.001). Compared with control group, the cases reported obviously higher proportion of family history of AIS (12.8% vs. 6.5%; *P* < 0.001). There was no difference of the distribution in living arrangement, academic pressure, enrollment pressure, classmate relationships and teacher-classmate relationships between cases and controls (all *Ps* > 0.05).

**Table 1:** Demographic characteristics of all participants.

**Variable**	**AIS, n (%)**	**Controls, n (%)**	*P*-value*
Total	491 (26.7)	1346 (73.3)	
Sex			0.970
Male	136 (27.7)	374 (27.8)	
Female	355 (72.3)	972 (72.2)	
Age (year), mean (SD)	13.69 (1.79)	13.81 (1.72)	0.178
BMI (kg/m^2^), mean (SD)	18.40 (2.80)	19.92 (3.49)	**<0.001**
Family history of AIS			**<0.001**
No	428 (87.2)	1258 (93.5)	
Yes	63 (12.8)	88 (6.5)	
Living arrangement			0.905
School dormitory	154 (31.7)	426 (32.0)	
Home	332 (68.3)	906 (68.0)	
Missing data	5	14	
Classmate relationships			0.060
Good	407 (83.2)	1145 (85.1)	
Average	73 (14.9)	193 (14.3)	
Poor	9 (1.8)	8 (0.6)	
Missing data	2	0	
Teacher-classmate relationships			0.693
Good	365 (74.6)	976 (72.7)	
Average	120 (24.5)	354 (26.4)	
Poor	4 (0.8)	13 (1.0)	
Missing data	2	3	
Academic pressure			0.957
None	15 (3.1)	44 (3.3)	
Low	383 (79.0)	1051 (78.4)	
High	87 (17.9)	246 (18.3)	
Missing data	6	5	
Enrollment pressure			0.072
None	12 (2.5)	44 (3.3)	
Low	333 (68.7)	844 (62.9)	
High	140 (28.9)	454 (33.8)	
Missing data	6	4	

Abbreviation: AIS, adolescent idiopathic scoliosis; BMI, body mass index; NA, not available

*The chi-square test was used for categorical variables and the *t*-test was used for age and BMI data.

### Univariable logistic regression analysis

Table 2 presents the results of the univariable logistic regression analysis of the association between academic-related factors and daily lifestyle habits and AIS. As for academic-related factors, our study indicated that individuals with poor reading and writing posture were more likely to have AIS (OR: 2.09, 95%CI: 1.67–2.61). In addition, the habit of crossing legs, the weight of the school bags and daily screen time on weekdays were also statistically associated with AIS (*all Ps* < 0.05). However, in this study, no significant associations between myopia, dominant hand when writing, mode of transportation of backpacks and AIS were observed (*all Ps* > 0.05).

**Table 2:** Associations between academic-related factors and daily lifestyle habits and AIS.

**Variable**	**AIS, n (%)**	**Controls, n (%)**	***OR* (95%*CI*)**	***P*-value**
**Academic-related factors**				
Myopia				0.637
Not	123 (25.1)	323 (24.0)	1.00 (reference)	
Yes	367 (74.9)	1021 (76.0)	0.94 (0.74, 1.20)	
Missing data	1	2	NA	
Reading and writing posture				**<0.001**
Good	143 (29.4)	626 (46.5)	1.00 (reference)	
Poor	344 (70.6)	720 (53.5)	2.09 (1.67, 2.61)	
Missing data	4	0	NA	
Dominant hand when writing				0.258
Left	12 (2.4)	22 (1.6)	1.00 (reference)	
Right	479 (97.6)	1324 (98.4)	0.66 (0.33, 1.35)	
Mode of transportation of backpacks				0.117
With both handles of the backpack on each shoulder	410 (83.5)	1078 (80.1)	1.00 (reference)	
With one of the handles on a shoulder or by a hand	76 (15.5)	260 (19.3)	0.77 (0.58, 1.02)	
Others	5 (1.0)	8 (0.6)	1.64 (0.54, 5.05)	
Weight of the school bags				**<0.001**
Light	59 (12.0)	176 (13.1)	1.00 (reference)	
Medium	257 (52.3)	915 (68.0)	0.84 (0.61, 1.16)	
Heavy	175 (35.6)	255 (18.9)	2.05 (1.44, 2.91)	
Cross your legs				**0.001**
Not	219 (44.6)	723 (53.7)	1.00 (reference)	
Yes	272 (55.4)	623 (46.3)	1.44 (1.17, 1.77)	
Screen time on a weekday				**<0.001**
≤ 2 h	427 (88.0)	1291 (96.8)	1.00 (reference)	
> 2 h	58 (12.0)	42 (3.2)	4.18 (2.77, 6.30)	
Missing data	6	13	NA	
**Daily lifestyle habits**				
Intake of milk and dairy products				**<0.001**
Yes	419 (85.5)	1242 (92.5)	1.00 (reference)	
Not	71 (14.5)	101 (7.5)	2.08 (1.51, 2.88)	
Missing data	1	3	NA	
Sleep quality (PSQI score)				0.409
Very good (0–5)	296 (60.8)	766 (56.9)	1.00 (reference)	
Okay (6–10)	176 (36.1)	517 (38.4)	0.88 (0.71, 1.10)	
Average (11–15)	15 (3.1)	59 (4.4)	0.66 (0.37, 1.18)	
Poor (16–21)	0 (0.0)	4 (0.3)	NA	
Missing data	4	0	NA	
Sleeping posture				**0.001**
Stretch	178 (36.9)	622 (46.3)	1.00 (reference)	
Curl up	232 (48.0)	539 (40.2)	1.50 (1.20, 1.89)	
Unclear	73 (15.1)	181 (13.5)	1.41 (1.03, 1.94)	
Missing data	8	4	NA	

Abbreviation: AIS, adolescent idiopathic scoliosis; 95%CI, 95% confidence interval; NA, not available

Furthermore, regarding daily lifestyle habits, compared to those who had the habit of taking milk and dairy products during last year, those who didn’t demonstrated a higher risk of developing AIS (OR: 2.08, 95%CI: 1.51–2.88). Adolescents with curled up sleeping posture seem to be more likely to experience AIS (OR: 1.50, 95%CI: 1.20–1.89).

### Multivariable logistic regression analysis

Multivariable logistic regression analysis was used to assess the associations between the factors that were statistical significant in univariable logistic regression analysis and AIS and the results was shown in Table 3. Model 1 included all academic and lifestyle factors that are significant in univariable analysis in Table 2, without adjusting for any confounding factors. Model 2 adjusted for BMI, living arrangement, family history of AIS, classmate relationships, teacher-classmate relationships, academic pressure and enrollment pressure. After adjusting for these confounding variables, adolescents with poor reading and writing posture were more likely to have AIS (AOR: 2.06, 95%CI: 1.58–2.68). Moreover, compared with those students carrying light school bags, those who reported that the weight of the school bags was heavy had higher risk for AIS (AOR: 2.22, 95%CI: 1.50–3.31). Furthermore, the daily screen time on weekdays more than 2 hours was significantly associated with AIS (AOR: 3.85, 95%CI: 2.43–6.11). Without the habit of taking milk and dairy products was also a risk factor for AIS (AOR: 1.87, 95%CI: 1.29–2.71).

**Table 3:** Multivariable logistic regression analysis with significant factors.

**Variable**	**Model 1**		**Model 2**	
	
	***OR* (95%*CI*)**	***P*-value**	***AOR* (95%*CI*)**	***P*-value**
**Academic-related factors**				
**Reading and writing posture** (Ref: Good)				
Poor	1.80 (1.41–2.30)	**<0.001**	2.06 (1.58–2.68)	**<0.001**
**Weight of the school bags** (Ref: Light)				
Medium	0.86 (0.61–1.20)	0.372	0.93 (0.65–1.34)	0.709
Heavy	2.05 (1.42–2.95)	**<0.001**	2.22 (1.50–3.31)	**<0.001**
**Screen time on a weekday** (Ref: ≤ 2 h)				
> 2 h	3.25 (2.10–5.03)	**<0.001**	3.85 (2.43–6.11)	**<0.001**
**Cross your legs** (Ref: Not)				
Yes	1.23 (0.98–1.55)	0.073	1.21 (0.95–1.55)	0.119
**Daily lifestyle habits**				
**Intake of milk and dairy products** (Ref: Yes)				
Not	1.81 (1.28–2.56)	**0.001**	1.87 (1.29–2.71)	**0.001**
**Sleeping posture** (Ref: Stretch)				
Curl up	1.20 (0.94–1.53)	0.146	1.08 (0.83–1.41)	0.556
Unclear	1.38 (0.99–1.93)	0.058	1.43 (1.00–2.05)	**0.048**

Abbreviations: 95% CI, 95% confidence interval; Ref, reference; AOR, adjusted OR

Model 1 unadjusted for confounding variables; Model 2 adjusted for BMI, living arrangement, family history of AIS, classmate relationships, teacher-classmate relationships, academic pressure and enrollment pressure.

## Discussion

The present study demonstrated that there were significant associations between academic-related factors and AIS. Poor reading and writing posture, heavy school bags and daily screen time on weekdays > 2 hours were significantly associated with AIS in adolescents. In addition, regarding daily lifestyle habits, the habit of intake of milk and dairy products was significantly associated with AIS.

The present study revealed that adolescents with incorrect reading and writing posture were at a higher risk for AIS. It has been proven in previous studies that incorrect posture was significantly associated with the onset and progress of AIS [41, 42], which could mainly be explained by the mechanical properties of the spine [43]. Adolescents are experiencing bone growth and are vulnerable to daily factors [44] and they always spend long periods of time in a sedentary, reading and writing state. The adoption of inappropriate reading and writing posture for long periods could lead to the fatigue and asymmetry of strength of the paravertebral muscles, thereby changing the biomechanical structure and affecting the shape and function of the spine [21, 41]. Therefore, it is very important to correct students’ postures and strengthen the paravertebral muscles.

Moreover, in our study, we found that heavy school bags were a significant risk factor of AIS. A similar result was obtained by Wang et al. [45] in junior middle school boys. In addition, previous studies have shown that backpack load could also affect AIS subjects’ balance [46] and pulmonary function [47]. Hence, reducing the weight of the student’s backpack or using a bag with wheels to reduce the burden on the spine is highly recommended.

Another variable related to AIS in the current study was the daily screen time on weekdays, including time spent on various electronic products such as computers, TVs, mobile phones, etc. It has been reported that excessive screen time, always accompanied by sedentary behaviour and a lack of physical activity, is harmful to the muscular fitness and bone health of adolescents [48]. Ciaccia MCC et al. [49] found that the prevalence of AIS was higher in students who adopted a sitting position for a long period of time. Due to the influence of COVID-19 pandemic, a lot of adolescents in Shenzhen take online classes using computers or tablets to complete their homework, which increased the screen time while reducing the time spent on outdoor activities. According to the recommended standard of “Chinese Children and Adolescent Physical Activity Guidelines”, daily ST for children and adolescents aged 6–17 should be limited to within 2 hours [50]. Therefore, in order to reduce the risk of AIS, we suggest that the amount of time spent on sedentary behavior (i.e. time spent on recreational screen activities) need to be restricted.

Regarding daily lifestyle habits, our current study indicated that adolescents who didn’t get used to intake of milk and dairy products were of higher risk for AIS. One possible explanation of this result was that milk and dairy products were rich in a variety of nutrients, such as protein, vitamin D, calcium, potassium, etc. These nutrients have been proven to be very beneficial for bone health and bone density, reducing the risk of osteopenia [29, 51]. A cross-sectional study in Japan involving 38,719 high school students showed that there was a positive dose-response relationship between milk intake and bone strength in late adolescents, and intake of milk 400 ml/day or more was beneficial for bone mass [29]. Rizzoli et al. [51] conducted a research on bone turnover and concluded that milk or dairy products are beneficial to bone health. Their study showed a significant negative correlation between dairy foods intake and bone turnover markers, and a positive correlation with bone mineral content. All these results indicated a dose-response relationship between bone density and milk or dairy products. Although the etiology of AIS was still unclear, it has been widely reported in previous researches that nearly 30% patients with AIS were found to be osteopenic [30, 31, 52], an important factor which is closely associated with the curve progression and disease prognosis. Milk is rich with minerals, especially calcium, which are of great significance to the healthy growth of children and adolescents. However, according to the current dietary structure of Chinese residents, the consumption of milk and dairy foods is at a low level [53]. Therefore, increasing the intake of milk and dairy in adolescents might be an important strategy for bone health promotion and AIS prevention.

There are some limitations of the present study that need to be highlighted. First, it was a case-control study, which didn’t allow us to confirm causal relationship. Second, we used a self-reported questionnaire to investigate past risk factors, so the recall bias was inevitable. Third, all participants in our study were adolescents in school, and those who were absent from school were not included. Last, physical activity was not taken into account in our study and this important factor will be considered in subsequent studies.

## Conclusions

In summary, academic-related factors and daily lifestyle habits were significantly associated with AIS among Chinese adolescents. Schools, families, and related facilities are recommended to take actions together to urge adolescent students to cultivate good academic-related behaviors and healthy lifestyle to maintain and promote spine health. However, longitudinal researches are needed in the future to explore the causal relationship between these lifestyle-related factors and AIS.

## Abbreviations

AIS: Adolescent idiopathic scoliosis
CI: Confidence interval
SD: Standard deviation
OR: Odds ratio
AOR: Adjusted odds ratio
CSSSP: Chinese school-based scoliosis screening program
SYSHC: Shenzhen youth spine health center
FBT: Forward bending test
ATR: Angle of trunk rotation
BMI: Body mass index
PSQI: Pittsburgh sleep quality index
ST: Screen time

## Declarations

### Availability of data and material

The raw data supporting the conclusions of the current study are available on reasonable request from the authors, without undue reservation.
